# Oral hygiene and the overall survival of head and neck cancer patients

**DOI:** 10.1002/cam4.2059

**Published:** 2019-03-13

**Authors:** Chan‐Chi Chang, Wei‐Ting Lee, Jenn‐Ren Hsiao, Chun‐Yen Ou, Cheng‐Chih Huang, Sen‐Tien Tsai, Ken‐Chung Chen, Jehn‐Shyun Huang, Tung‐Yiu Wong, Yu‐Hsuan Lai, Yuan‐Hua Wu, Wei‐Ting Hsueh, Shang‐Yin Wu, Chia‐Jui Yen, Jang‐Yang Chang, Chen‐Lin Lin, Ya‐Ling Weng, Han‐Chien Yang, Yu‐Shan Chen, Jeffrey S. Chang

**Affiliations:** ^1^ Department of Otolaryngology National Cheng Kung University Hospital, College of Medicine, National Cheng Kung University Tainan Taiwan; ^2^ Institute of Clinical Medicine, College of Medicine, National Cheng Kung University Tainan Taiwan; ^3^ Department of Stomatology National Cheng Kung University Hospital, College of Medicine, National Cheng Kung University Tainan Taiwan; ^4^ Department of Radiation Oncology National Cheng Kung University Hospital, College of Medicine, National Cheng Kung University Tainan Taiwan; ^5^ Division of Hematology/Oncology, Department of Internal Medicine National Cheng Kung University Hospital, College of Medicine, National Cheng Kung University Tainan Taiwan; ^6^ National Institute of Cancer Research, National Health Research Institutes Tainan Taiwan; ^7^ Department of Nursing National Cheng Kung University Hospital, College of Medicine, National Cheng Kung University Tainan Taiwan

**Keywords:** epidemiology and prevention, gene–environmental interaction, head and neck cancer, survival

## Abstract

Poor oral hygiene is an established risk factor of head and neck cancer (HNC); however, its role in the survival of HNC patients is unclear. This study evaluated the association between oral hygiene habits, including regular dental visits, frequency of tooth brushing, and use of dental floss, and the overall survival (OS) of HNC patients using interview data collected from 740 HNC patients. In addition, the interactions between oral hygiene and the polymorphisms of *TLR2 *and *TLR4* on the OS of HNC patients were assessed. The analysis indicated that poor oral hygiene was significantly associated with poorer OS of HNC patients (hazard ratio (HR) = 1.38, 95% confidence interval (CI): 1.03‐1.86). This association was modified by a single nucleotide polymorphism, rs11536889, of *TLR4*. A significant association between poor oral hygiene and worse survival of HNC was observed among those with the CG or CC genotype (HR = 2.32, 95% CI: 1.41‐3.82) but not among those with the GG genotype (HR = 0.95, 95% CI: 0.65‐1.40). Our results suggested that poor oral hygiene is not only a risk factor but may also be a prognostic factor of HNC.

## Abstract 2

Poor oral hygiene was associated with a worse survival of head and neck cancer. TLR4 affected the survival of head and neck cancer due to poor oral hygiene. Poor oral hygiene is both a risk and a prognostic factor of head and neck cancer

## 1 INTRODUCTION

Head and neck cancer (HNC), including cancers of the oral cavity, oropharynx, hypopharynx, and larynx, is the fifth leading cancer in the world, with approximately 600 000 new cases diagnosed worldwide annually^1^ Alcohol, betel quids, and cigarettes are the major risk factors of HNC and contribute to the majority of the HNC cases.^2^ In addition, rising trends in the incidence of human papillomavirus–associated oropharyngeal cancer have been reported, particularly in the Western countries.^3^

In addition to the above mentioned risk factors, recent studies have generated consistent results to establish poor oral hygiene as an independent risk factor of HNC. A meta‐analysis of 18 case–control studies showed that lower frequency of tooth brushing was associated with an increased HNC risk (odds ratio [OR] = 2.08, 95% confidence interval [CI]: 1.65‐2.62).^4^ The majority of published studies reported an increased HNC risk associated with no regular dental visit.^5^, ^6^ In a case–control study of 374 cases and 374 controls, Elwood et al^5^ reported that no regular dental care was associated with a 1.6 times (relative risk [RR] = 1.6, *P* < 0.05) increase in HNC risk. Marshall et al^6^ conducted a case–control study of 290 oral cancer cases and 290 controls and found that no regular dental check‐ups was associated with a statistically significant increase in oral cancer risk, although no dose–response relationship was observed. In a study of 122 oral and pharyngeal cancer patients and 124 controls conducted by Lissowaska et al,^7^ individuals who never had a dental check‐up had a 12 times increase in the risk of oral and pharyngeal cancer (OR = 11.89, 95% CI: 3.33‐42.51). In a study of 132 patients with oral and oropharyngeal cancer and 320 controls, Rosenquist et al^8^ showed that having a regular dental check‐up was associated with a decreased risk of oral and oropharyngeal cancer (OR = 0.4, 95% CI: 0.2‐0.6). In a large multicenter case–control study, which included 2286 HNC patients and 1824 controls from Latin America, never having any dental check‐up was associated with a significantly elevated HNC risk (OR = 1.61, 95% CI: 1.18‐2.20).^9^ In a large population–based case–control study with 1361 HNC cases and 1289 controls, Divaris et al^10^ found that routine dental visits were associated with an approximately 30% reduction in HNC risk (OR = 0.68, 95% CI: 0.53‐0.87). Using data from a case–control study of 317 HNC patients and 296 controls, Chang et al^11^ found that having no regular dental visits was associated with an almost three times increase in HNC risk (OR = 2.86, 95% CI: 1.47‐5.57). Ahrens et al found that in a case–control study of 1963 patients with upper aerodigestive tract cancer (HNC + esophageal cancer) and 1993 controls, those who never visited the dentist had almost two times increase in the risk of upper aerodigestive tract cancer (OR = 1.93, 95% CI: 1.48‐2.51).^12^ In a case–control study pooling data from 8925 HNC cases and 12 527 controls, Hashim et al^13^ showed that visiting the dentist at least once per year was associated with a reduced HNC risk (OR = 0.78, 95% CI: 0.72‐0.85). Poor oral hygiene may result in periodontal diseases, which were associated with an increased HNC risk (OR = 2.63, 95% CI: 1.68‐4.14) according to a meta‐analysis of two cohort and six case–control studies.^14^

Although the role of poor oral hygiene in the risk of HNC is well recognized, its influence on the prognosis of HNC is unclear. Only two studies to date have examined the association between oral hygiene and the prognosis of HNC. The study by Farquhar et al^15^ observed that >10 dental visits in the past 10 years was associated with a decreased mortality for HNC patients (hazard ratio [HR] = 0.6, 95% CI: 0.4‐0.8) and this association was particularly strong for oral cancer (HR = 0.4, 95% CI: 0.2‐0.9). Friemel et al^16^ reported that poor dental care was associated with a poorer overall survival (OS) of HNC, but the result was not statistically significant (HR = 1.30, 95% CI: 0.78‐2.15).

Because of the limited information regarding the role of oral hygiene in the prognosis of HNC, we investigated the association between oral hygiene habits and the survival of HNC patients. Furthermore, poor oral hygiene may promote the growth of periodontopathogenic bacteria, which may bind to toll–like receptors (TLRs) to induce inflammation.^17^ Inflammation indicated by higher levels of inflammatory markers has been associated with poorer outcomes of HNC patients.^18^, ^19^ For this reason, we also evaluated the interaction between oral hygiene and the polymorphisms of the *TLR* genes on the survival of HNC patients.

## 2 MATERIALS AND METHODS

The institutional review boards of the National Health Research Institutes and the National Cheng Kung University Hospital approved the content and the execution of this study. The purpose of the study and the possible risk of participating in the study were explained to each potential study participant at the time of recruitment. A signed informed consent was obtained from each individual who agreed to join the study.

### 2.1 Study subject recruitment

This analysis included HNC cases recruited by an ongoing HNC case–control study that commenced subject recruitment on 1 September 2010 in the Department of Otolaryngology and the Department of Stomatology at the National Cheng Kung University Hospital. To be eligible for participating in the study, the subject needed to have or be: (a) diagnosis of pathologically confirmed squamous cell carcinoma of the head and neck, including cancers of the oral cavity, oropharynx, hypopharynx, and larynx (ICD‐10 codes: C00‐C10, C12‐C14, C32); (b) no previous diagnosis of any cancer; (c) aged 20 years or older; and (d) the ability to understand the purpose of the study and to give informed consent. Although we did not formally assess the performance status, HNC patients who were not physically or mentally stable to be interviewed were excluded from the study.

### 2.2 Interview survey data collection

A trained interviewer used a standardized questionnaire to conduct an in–person interview with each study participant to collect data on oral hygiene habits. The oral hygiene habits included: (a) regular dental visits (yes/no and frequency); (b) tooth brushing (number of times per day); and (c) use of dental floss (yes/no).

Data on potential confounders, including sex, age, education, and use of alcohol, betel quids and cigarettes, were also collected.

### 2.3 Clinical information and vital status

The cancer registry of the National Cheng Kung University Hospital was searched to obtain the clinical information, including cancer stage (AJCC staging, seventh edition) and treatment modality, and the vital status of the HNC patients. The oncology case manager maintains and regularly updates the clinical information in the hospital cancer registry according to information in the medical records. The vital status in the hospital tumor registry was recorded according to three sources: (a) regular updates of vital status provided by the Health Promotion Administration of Taiwan; (b) hospital medical records; (c) active follow‐up of patients by oncology case managers. For this analysis, patients were followed until 12 July 2017.

### 2.4 Collection of blood samples

A pretreatment blood sample was drawn from each study participant and collected in an EDTA–containing vacutainer tube. The blood sample was first centrifuged to separate out the buffy coat. A commercially available DNA purification kit was then used to extract DNA from the buffy coat. The DNA samples were stored in a −80°C refrigerator until use for genotyping.

### 2.5 Selection of TLR genes and SNPs

Since poor oral hygiene may promote the growth of periodontopathogenic bacteria, which may bind to toll–like receptors (TLRs), particularly TLR2 and TLR4, to induce inflammation 17, 20, we decided to select single nucleotide polymorphisms (SNPs) of *TLR2* and *TLR4*. We first conducted extensive literature review to select the *TLR2* and *TLR4* SNPs commonly investigated in cancer association studies. From the literature search we selected three SNPs for *TLR2*: rs5743708 (Arg753Gln), rs3804099, and rs3804100 and three SNPs for *TLR4*: rs4986791 (Thr399Ile), rs4986790 (Asp299Gly), and rs11536889. We then examined the minor allele frequencies of these SNPs in The Single Nucleotide Polymorphism database (dbSNP) (https://www.ncbi.nlm.nih.gov/snp/) and excluded those SNPs (*TLR2*: rs5743708 and *TLR4*: rs4986791 and rs4986790) with a minor allele frequency <5% in the HapMap Han Chinese population. The remaining three SNPs (*TLR2*: rs3804099, and rs3804100; *TLR4*: rs11536889) were included for further investigation.

### 2.6 Genotyping

Genotyping of two *TLR2* SNPs, rs3804099 and rs3804100, and one *TLR4* SNP, rs11536889, was accomplished with Taqman–based allelic discrimination method on an Applied Biosystems 7500 Real–Time Polymerase Chain Reaction System (Applied Biosystems, Foster City, CA). To detect genotyping errors, 10% of the samples were randomly selected for duplicate genotyping and a concordance of 100% was observed.

### 2.7 Statistical analysis

Cox proportional hazards models were constructed to assess the influence of oral hygiene habits on the OS of HNC patients. For individuals who died during the follow‐up period, the time of follow‐up was calculated from the date of HNC diagnosis to the date of death. For individuals who were still alive at the end of follow‐up, the follow‐up time was censored on the last date of the follow‐up on 12 July 2017.

The oral hygiene habits (regular dental visit, frequency of tooth brushing, and use of dental floss) were first analyzed separately for their influence on the OS of HNC patients. Subsequently, the three oral hygiene habits were combined to generate an oral hygiene score. The oral hygiene score = regular dental visit + frequency of tooth brushing + use of dental floss: regular dental visit: yes = 0, no = 1; frequency of tooth brushing: ≧ 2 times per day = 0, < 2 times per day = 1; and use of dental floss: yes = 0, no = 1. An oral hygiene score of 0 or 1 = good oral hygiene, 2 = moderate oral hygiene, and 3 = poor oral hygiene.

The associations between the potential confounders, including age, sex, education, use of alcohol, betel quids, and cigarettes, HNC stage, HNC grade, and HNC treatment modalities (surgery, radiation, and chemotherapy), and the OS of HNC patients were assessed using univariate Cox proportional hazards models. Alcohol use, HNC stage, HNC grade, and HNC treatment were significantly (*P* < 0.05) associated with the OS of HNC patients and were thus included in the final multivariate models examining the association between oral hygiene habits and the OS of HNC patients. Inclusion of age, sex, education, and use of cigarettes and betel quids in the multivariable models did not change the HRs of oral hygiene habits by more than 10% and were thus excluded from the final multivariable models.

The association between oral hygiene habits and the OS of HNC patients was first evaluated with all subsites of HNC combined and then by each subsite (oral cavity, oropharynx, hypopharynx, and larynx) separately to assess whether the association between oral hygiene and the OS of HNC patients might vary with the different subsites of HNC.

Since alcohol use was significantly associated with worse OS of HNC patients, we evaluated whether poor oral hygiene might interact with alcohol use synergistically to influence the OS of HNC patients. An interaction term (oral hygiene x alcohol use) was included in the Cox proportional hazards model. The significance of the interaction term was assessed by comparing the model with the interaction term to the model without the interaction term using log–likelihood ratio test.

To evaluate the influence of *TLR* genes on the association between oral hygiene habits and the OS of HNC patients, Cox proportional hazards models were performed stratified by the genotypes of the two *TLR2* SNPs (rs3804099 and rs3804100) and one *TLR4* SNP (rs11536889). The significance of the interaction term (oral hygiene × rs3804099 or oral hygiene × rs3804100 or oral hygiene × rs11536889) was evaluated by comparing the model with the interaction term to the model without the interaction term using log–likelihood ratio test.

## 3 RESULTS

A total of 740 HNC patients (467 oral cancers, 105 oropharyngeal cancers, 71 hypopharyngeal cancers, 86 laryngeal cancers, and 11 cancers of multiple HNC subsites) were included in the current analysis. Ninety‐four percent of the HNC patients were men (Table 1). Less than half of the subjects had completed high school education. The majority of the subjects were users of alcohol, betel quids and cigarettes. Fifty‐five percent of the HNC cases were diagnosed at stage 3 or 4. Fifty‐eight percent of the HNC cases had a moderate or high histologic grade. Sixty‐seven percent, 49%, and 41% of the patients had surgery, radiation, and chemotherapy, respectively.

**Table 1:** The association between demographic characteristics, lifestyle factors, and clinical characteristics and the overall survival of head and neck cancer patients

	N = 740 n (%)	HR (95% CI)	*P*
Age, mean (SE)	55.19 (0.39)	0.997 (0.984‐1.011)	0.68
Sex			
Men	693 (93.6)	Reference	
Women	47 (6.4)	0.64 (0.32‐1.31)	0.22
Education			
≦Elementary school	204 (27.6)	Reference	
Junior high	217 (29.3)	0.76 (0.53‐1.11)	0.15
High school/Technical school	242 (32.7)	0.81 (0.56‐1.16)	0.24
Some college or more	77 (10.4)	0.68 (0.39‐1.19)	0.18
Cigarette smoking			
Never	108 (14.6)	Reference	
Former	139 (18.8)	1.17 (0.67‐2.03)	0.58
Current	492 (66.6)	1.46 (0.92‐2.32)	0.10
Alcohol			
Never + occasional	239 (32.3)	Referent	
Former regular	99 (13.4)	1.78 (1.14‐2.79)	0.01
Current regular	402 (54.3)	1.38 (0.98‐1.94)	0.07
Betel quid chewing			
Never	222 (30.0)	Reference	
Former	280 (37.8)	1.00 (0.70‐1.43)	1.00
Current	238 (32.2)	1.11 (0.77‐1.59)	0.57
Stage			
1, 2	300 (40.5)	Reference	
3, 4	405 (54.7)	4.35 (2.95‐6.41)	<0.0001
Unknown	35 (4.7)	2.57 (1.18‐5.58)	0.02
Grade			
Low	229 (31.0)	Reference	
Moderate	338 (45.7)	1.53 (1.04‐2.25)	0.03
High	91 (12.3)	2.59 (1.62‐4.14)	<0.0001
Unknown	82 (11.1)	3.23 (2.05‐5.09)	<0.0001
Surgery			
No	232 (31.3)	Reference	
Yes	495 (66.9)	0.35 (0.26‐0.46)	<0.0001
Unknown	13 (1.8)	0.38 (0.09‐1.54)	0.18
Radiation			
No	352 (47.6)	Reference	
Yes	359 (48.5)	2.88 (2.08‐3.97)	<0.0001
Unknown	29 (3.9)	1.47 (0.63‐3.43)	0.37
Chemotherapy			
No	412 (55.7)	Reference	
Yes	304 (41.1)	3.53 (2.58‐4.83)	<0.0001
Unknown	24 (3.2)	2.04 (0.88‐4.72)	0.10

One hundred eighty‐six deaths (25.1%) occurred among the 740 HNC patients during the follow‐up. The median follow‐up time was 3.1 years. The OS of HNC patients was not significantly (*P* > 0.05) associated with age, sex, education, and use of cigarettes and betel quids (Table 1). Factors associated with poorer OS of HNC patients included alcohol use, advanced stages (stages 3 and 4), higher histologic grades, and treatment with radiation or chemotherapy. A better OS was observed for HNC patients who underwent surgery.

HNC patients who did not have regular dental visits had a worse OS compared to those with regular dental visits (HR = 2.05, 95% CI: 1.04‐4.04) (Table 2). No significant association was observed between OS of HNC patients and the frequency of tooth brushing and use of dental floss. HNC patients with poor oral hygiene (oral hygiene score = 3) had a worse OS compared to those with good or moderate oral hygiene (HR = 1.38, 95% CI: 1.03‐1.86).

**Table 2:** The association between oral hygiene habits and the overall survival of head and neck cancer patients

Oral hygiene habits	n (%)	Univariate HR (95% CI)	*P*	Multivariate HR (95% CI)^a^	*P*
Regular dental visits					
Yes	63 (8.5)	Reference		Reference	
No	677 (91.5)	1.89 (0.97‐3.70)	0.06	2.05 (1.04‐4.04)	0.04
Tooth brushing					
2 or more times per day	388 (52.6)	Reference		Reference	
<2 times per day	349 (47.4)	1.29 (0.97‐1.72)	0.08	1.23 (0.92‐1.65)	0.16
Use of dental floss					
Yes	184 (24.9)	Reference		Reference	
No	555 (75.1)	1.24 (0.87‐1.76)	0.24	1.17 (0.82‐1.67)	0.39
Oral hygiene score^b^					
1 (Good)	156 (21.2)	Reference		Reference	
2 (Moderate)	302 (41.0)	1.05 (0.69‐1.59)	0.82	1.01 (0.66‐1.54)	0.97
3 (Poor)	279 (37.9)	1.48 (0.99‐2.22)	0.05	1.39 (0.92‐2.09)	0.12
3 vs 1 + 2		1.44 (1.08‐1.92)	0.01	1.38 (1.03‐1.86)	0.03

a HR and 95% CI were calculated using Cox proportional hazards model, adjusted for alcohol use, stage, grade, surgery, radiation, and chemotherapy.

b Oral hygiene score = tooth brushing + use of dental floss + regular dental visit, with tooth brushing: ≧2 times per day = 0, <2 times per day = 1; Use of dental floss: yes = 0, no = 1; and regular dental visit: yes = 0, no = 1.

In the analysis stratified by the subsites of HNC, poor oral hygiene was associated with a borderline worse OS (*P* = 0.06) of oral cancer patients compared to those with moderate or good oral hygiene (Table 3). Infrequent tooth brushing (<2 times per day) and poor oral hygiene were significantly associated with a worse survival among laryngeal cancer patients. Poor oral hygiene was not associated with OS among patients with oropharyngeal or hypopharyngeal cancer.

**Table 3:** The association between oral hygiene habits and the overall survival of head and neck cancer by subsites

Oral hygiene habits	Oral cancer			Oropharyngeal cancer			Hypopharyngeal cancer			Laryngeal cancer		
	n (%)	HR (95% CI)^a^	*P*	n (%)	HR (95% CI)^a^	*P*	n (%)	HR (95% CI)^a^	*P*	n (%)	HR (95% CI)^a^	*P*
Regular dental visits												
Yes	40 (8.6)	Reference		13 (12.4)	Reference		3 (4.2)	Reference		7 (8.1)	Reference	
No	427 (91.4)	1.18 (0.50‐2.77)	0.71	92 (87.6)	3.34 (0.78‐14.25)	0.10	68 (95.8)	—	—	79 (91.9)	0.93 (0.11‐7.82)	0.94
Tooth brushing												
2 or more times per day	257 (55.2)	Reference		57 (54.3)	Reference		25 (35.7)	Reference		45 (52.9)	Reference	
<2 times per day	209 (44.9)	1.33 (0.88‐2.01)	0.17	48 (45.7)	0.95 (0.44‐2.05)	0.90	45 (64.3)	0.62 (0.27‐1.42)	0.26	40 (47.1)	3.76 (1.11‐12.80)	0.03
Use of dental floss												
Yes	123 (26.3)	Reference		25 (23.8)	Reference		19 (27.1)	Reference		15 (17.4)	Reference	
No	344 (73.7)	1.19 (0.73‐1.93)	0.49	80 (76.2)	0.75 (0.33‐1.72)	0.50	51 (72.9)	1.80 (0.66‐4.92)	0.25	71 (82.6)	8.54 (0.35‐206.33)	0.19
Oral hygiene score^b^												
1 (Good)	102 (21.9)	Reference		26 (24.8)	Reference			Reference		16 (18.8)	Reference	
2 (Moderate)	201 (43.1)	0.88 (0.50‐1.54)	0.66	39 (37.1)	1.68 (0.70‐4.06)	0.25	11 (15.7)	0.61 (0.17‐2.22)	0.46	34 (40.0)	0.43 (0.04‐4.48)	0.48
3 (Poor)	163 (35.0)	1.36 (0.78‐2.38)	0.28	40 (38.1)	1.09 (0.40‐2.97)	0.87	24 (34.3)	0.84 (0.27‐2.59)	0.76	35 (41.2)	3.11 (0.47‐20.44)	0.24
3 vs 1 + 2		1.48 (0.98‐2.24)	0.06		0.80 (0.35‐1.84)	0.60	35 (50.0)	1.17 (0.54‐2.53)	0.69		5.35 (1.46‐19.60)	0.01

a HR and 95% CI were calculated using Cox proportional hazards model, adjusted for alcohol use, stage, grade, surgery, radiation, and chemotherapy.

b Oral hygiene score = tooth brushing + use of dental floss + regular dental visit, with tooth brushing: ≧2 times per day = 0, <2 times per day = 1; Use of dental floss: yes = 0, no = 1; and regular dental visit: yes = 0, no = 1.

Our results indicated no significant interaction (*P*‐interaction >0.05) between alcohol use and oral hygiene habits on the OS of HNC patients (Table 4). The HRs suggested that poor oral hygiene was associated with worse OS of HNC patients regardless of the alcohol drinking status; however, the HRs were all nonstatistically significant likely due to the smaller sample size in each stratum of the alcohol drinking status.

**Table 4:** The association between oral hygiene habits and overall survival of head and neck cancer patients by alcohol drinking status

Oral hygiene habits	Never + occasional drinkers			Ever regular drinkers		
	n (%)	HR (95% CI)^a^	*P*	n (%)	HR (95% CI)^a^	*P*
Regular dental visits						
Yes	31 (13.0)	Reference		32 (6.4)	Reference	
No	208 (87.0)	2.03 (0.71‐5.80)	0.18	469 (93.6)	1.88 (0.77‐4.61)	0.16
	*P*‐interaction = 0.81					
Tooth brushing						
2 or more times per day	144 (60.5)	Reference		244 (51.1)	Reference	
<2 times per day	94 (39.5)	1.58 (0.87‐2.87)	0.13	255 (48.9)	1.15 (0.82‐1.61)	0.43
	*P*‐interaction = 0.49					
Use of dental floss						
Yes	71 (29.7)	Reference		113 (22.6)	Reference	
No	168 (70.3)	1.22 (0.61‐2.44)	0.57	387 (77.4)	1.23 (0.80‐1.89)	0.33
	*P*‐interaction = 0.69					
Oral hygiene score^b^						
1 (Good)	70 (29.4)	Reference		86 (17.2)	Reference	
2 (Moderate)	95 (39.9)	1.04 (0.49‐2.20)	0.93	207 (41.5)	1.02 (0.61‐1.70)	0.95
3 (Poor)	73 (30.7)	1.89 (0.88‐4.04)	0.10	206 (41.3)	1.31 (0.80‐2.15)	0.29
	*P*‐interaction = 0.81					
3 vs 1 + 2		1.85 (0.99‐3.47)	0.06		1.29 (0.92‐1.82)	0.13
	*P*‐interaction = 0.54					

a HR and 95% CI were calculated using Cox proportional hazards model, adjusted for stage, grade, surgery, radiation, and chemotherapy.

b Oral hygiene score = tooth brushing + use of dental floss + regular dental visit, with tooth brushing: ≧2 times per day = 0, <2 times per day = 1; Use of dental floss: yes = 0, no = 1; and regular dental visit: yes = 0, no = 1.

The two *TLR2* SNPs, rs3804099 and rs3804100, and *TLR4 *rs11536889 were not significantly associated with the OS of HNC patients (Table 5). TLR2 rs3804099 and rs3804100 did not modify the relationship between poor oral hygiene and the OS of HNC patients (*P*‐interaction >0.05) (Table 6). In contrast, the association between oral hygiene and the OS of HNC patients differed according to the genotype of *TLR4 *rs11536889. Tooth brushing less than two times per day was associated with a worse survival among HNC patients with the CG or CC genotype (HR = 1.97, 95% CI: 1.20‐3.22) but not among those with the GG genotype (HR = 0.89, 95% CI: 0.61‐1.30) (*P*‐interaction = 0.04). Similarly, among HNC patients with the CG or CC genotype , poor oral hygiene was associated with a worse OS (HR = 2.32, 95% CI: 1.41‐3.82), whereas among HNC patients with the GG genotype, poor oral hygiene showed no association with the OS (HR = 0.95, 95% CI: 0.65‐1.40) (*P*‐interaction = 0.02).

**Table 5:** the association between genetic polymorphisms of *TLR2* and *TLR4 *and the overall survival of head and neck cancer patients

*TLR* polymorphisms	n (%)	HR (95% CI)^a^	P
*TLR2 *rs3804099			
TT	343 (47.5)	Reference	
CT	323 (44.7)	1.13 (0.83‐1.53)	0.45
CC	56 (7.8)	0.83 (0.47‐1.44)	0.50
CT+CC		0.94 (0.70‐1.26)	0.66
*TLR2 *rs3804100			
TT	377 (52.2)	Reference	
CT	298 (41.3)	1.26 (0.93‐1.70)	0.14
CC	47 (6.5)	0.59 (0.29‐1.17)	0.13
TT+CC		0.88 (0.65‐1.18)	0.39
*TLR4 *rs11536889			
GG	422 (58.6)	Reference	
CG	249 (34.6)	0.80 (0.58‐1.12)	0.19
CC	49 (6.8)	0.70 (0.38‐1.27)	0.24
CG+CC		0.78 (0.58‐1.07)	0.12

a HR and 95% CI were calculated using Cox proportional hazards model, adjusted for dental care habits, alcohol use, stage, grade, surgery, radiation, and chemotherapy.

**Table 6:** The association between oral hygiene habits and the overall survival of head and neck cancer patients by genetic polymorphisms of *TLR2 *and *TLR4*

Oral hygiene habits	*TLR2 *rs3804099		*TLR2 *rs3804100		*TLR4 *rs11536889	
	TT	CT+CC	TT	CT+CC	GG	CG+CC
	HR (95% CI)^a^	HR (95% CI)^a^	HR (95% CI)^a^	HR (95% CI)^a^	HR (95% CI)^a^	HR (95% CI)^a^
Regular dental visits						
Yes	Reference	Reference	Reference	Reference	Reference	Reference
No	2.21 (0.88‐5.53)	1.54 (0.54‐4.34)	1.66 (0.66‐4.16)	2.09 (0.74‐5.85)	1.55 (0.56‐4.27)	1.93 (0.76‐4.90)
	*P*‐interaction = 0.70		*P*‐interaction = 0.77		*P*‐interaction = 0.79	
Tooth brushing						
2 or more times per day	Reference	Reference	Reference	Reference	Reference	Reference
<2 times per day	1.26 (0.84‐1.88)	1.06 (0.68‐1.67)	1.21 (0.80‐1.84)	1.14 (0.74‐1.76)	0.89 (0.61‐1.30)	1.97 (1.20‐3.22)
	*P*‐interaction = 0.67		*P*‐interaction = 0.77		*P*‐interaction = 0.04	
Use of dental floss						
Yes	Reference	Reference	Reference	Reference	Reference	Reference
No	0.93 (0.58‐1.50)	1.57 (0.90‐2.74)	1.01 (0.61‐1.67)	1.37 (0.81‐2.33)	0.94 (0.60‐1.45)	1.56 (0.80‐3.03)
	*P*‐interaction = 0.23		*P*‐interaction = 0.45		*P*‐interaction = 0.14	
Oral hygiene score^b^						
1 (Good)	Reference	Reference	Reference	Reference	Reference	Reference
2 (Moderate)	1.13 (0.64‐2.00)	0.90 (0.47‐1.70)	1.08 (0.59‐1.96)	0.94 (0.51‐1.72)	0.92 (0.55‐1.55)	0.95 (0.44‐2.02)
3 (Poor)	1.38 (0.79‐2.41)	1.26 (0.67‐2.35)	1.30 (0.72‐2.33)	1.34 (0.74‐2.41)	0.90 (0.54‐1.50)	2.24 (1.09‐4.59)
	*P*‐interaction = 0.85		*P*‐interaction = 0.89		*P*‐interaction = 0.06	
3 vs 1 + 2	1.28 (0.85‐1.91)	1.35 (0.86‐2.14)	1.23 (0.81‐1.88)	1.40 (0.90‐2.17)	0.95 (0.65‐1.40)	2.32 (1.41‐3.82)
	*P*‐interaction = 0.86		*P*‐interaction = 0.78		*P*‐interaction = 0.02	

a HR and 95% CI were calculated using Cox proportional hazards model, adjusted for alcohol use, stage, grade, surgery, radiation, and chemotherapy.

b Oral hygiene score = tooth brushing + use of dental floss + regular dental visit, with tooth brushing: ≧2 times per day = 0, <2 times per day = 1; Use of dental floss: yes = 0, no = 1; and regular dental visit: yes = 0, no = 1.

## 4 DISCUSSION

In the current study, we found that a lack of regular dental visits and overall poor oral hygiene (oral hygiene score = 3) were associated with a worse survival of HNC patients. This association was modified by *TLR4 *rs11536889 with the worse survival associated with poor oral hygiene observed among HNC patients with the CG or CC genotype but not among HNC patients with the GG genotype.

Our result is consistent with results from the only two previous studies that examined the association between oral hygiene and the survival of HNC patients^15^, ^16^. Similar to our study, Friemel et al used a composite dental care score that consisted of tooth brushing, use of dental floss, and dentist visits, and they found that poor dental care was associated with a poorer OS of HNC, but the result did not reach statistical significance (HR = 1.30, 95% CI: 0.78‐2.15). The nonstatistical significance could be due to a lower statistical power associated with the smaller sample size (n = 263). Farquhar et al analyzed the data of 1381 HNC patients and found that >10 dental visits in the past 10 years was associated with a reduced mortality for HNC patients (HR = 0.6, 95% CI: 0.4‐0.8), particularly for those with oral cancer (HR = 0.4, 95% CI: 0.2‐0.9).^15^ These two studies together with our study suggested that poor oral hygiene may be associated with a poorer survival of HNC patients. However, due to the limited number of studies, more investigations are needed to confirm these findings.

Poor oral hygiene may result in the overgrowth of pathogenic bacteria in the oral cavity. These pathogenic bacteria may induce inflammation^17^ Inflammation in turn may lead to poorer survival among HNC patients.^18^, ^19^ In addition to inducing inflammation, pathogenic bacteria may promote the progression of HNC through other mechanisms. *Fusobacterium nucleatum* a well–known species of periodontopathogenic bacteria, has been shown to promote cell proliferation and increase cellular migration and invasion,^21^ and thus has a potential to promote the progression of HNC. To determine the biological mechanisms underlying the association between poor oral hygiene and the decreased survival of HNC patients, future studies need to focus on the role of microbiome in the prognosis of HNC.

Another possible explanation for the association between poor oral hygiene, particularly no regular dental visits, and a poorer prognosis of HNC is that HNC patients who had visited dentists regularly might be more likely to be diagnosed at an earlier stage. This was supported by our results showing that HNC patients who had regular dental visits were more likely to be diagnosed with early T‐stages, which consisted of smaller tumor sizes (Supplementary Table S1). However, even after adjusting for tumor stage, poor oral hygiene remained significantly associated with a poorer survival of HNC patients, suggesting that poor oral hygiene is a prognostic indicator for HNC survival, independent of the tumor stage.

In the subsite analysis, we found that poor oral hygiene was associated with a borderline worse OS (*P* = 0.06) among oral cancer patients. In addition, tooth brushing <2 times per day and poor oral hygiene (oral hygiene score = 3) were significantly associated with a worse OS among laryngeal cancer patients. The only other study that examined the association between oral hygiene and HNC survival by subsite reported that >10 dental visits in the past 10 years was associated with a reduced mortality most significantly for oral cancer followed by oropharyngeal cancer and least associated with the survival among patients with laryngeal or hypopharyngeal cancer.^15^ Because of the proximity, it was not surprising to see the influence of oral hygiene on the survival of oral cancer patients. It was unclear why we saw an even stronger association between poor oral hygiene and the worse survival among laryngeal cancer patients. Chance finding could not be ruled out because of the smaller sample size for each subsite of the HNC.

Our results showed that *TLR4 *rs11536889 modified the relationship between oral hygiene and the OS of HNC patients. Poor oral hygiene was associated with a significantly worse OS among HNC patients with the CG or CC genotype but not among HNC patients with the GG genotype. We further examined the linkage structure of the SNPs with a minor allele frequency ≧5% on the *TLR4* gene using Han Chinese data from the HapMap database. The linkage structure was analyzed with Haploview, version 4.2.^22^ The linkage structure showed that rs11536889 was not linked (r^2^<0.8) to any other SNPs of the *TLR4* gene (Supplementary Figure S1), indicating that the gene–environment interaction between poor oral hygiene and rs11536889 was a true signal and not marking the contribution of another SNP. Furthermore, a laboratory study by Sato et al revealed the function of rs11536889.^23^ Rs11536889 is a functional SNP residing in the 3′‐untranslated region of *TLR4*. Higher levels of TLR4 expressed by the peripheral blood monocytes were found among individuals with the rs11536889 CC genotype compared to those with the GC or GG genotype.^23^ Further investigation revealed that a binding site for two micro RNAs, hsa‐miR‐1236 and has‐miR‐642a, was created by the G allele, and the binding of the micro RNAs down‐regulated the level of TLR4^23^ The CC genotype of rs11536889 was associated with an increased risk of moderate and chronic periodontitis,^24^ indicating that the C allele is associated with a tendency to develop inflammation. This also suggested that a lower level of TLR4 among individuals with the rs11536889 GG genotype may generate a milder inflammatory process in response to periodontopathogenic bacteria infection, which is often the result of poor oral hygiene. Altogether, they suggested that poor oral hygiene may reduce the survival of HNC patients through the inflammatory pathways, although more investigations are needed to corroborate this hypothesis.

This study has several limitations. First, the subsite analysis might have suffered from the lack of statistical power due to the smaller sample size for each subsite. In addition to the lack of statistical power, a smaller sample size in the stratified analyses may increase the probability of chance findings. Second, we did not have access to the tumor tissue to test for the HPV infection status and thus we could not adjust for the influence of HPV in our statistical models. Studies have shown that HPV infection plays a minimal role in the development of nonoropharyngeal HNC^25^ and therefore not adjusting for HPV status likely only had an impact for oropharyngeal cancer. Poor oral hygiene has been positively associated with oral HPV infection.^26^ Given the more favorable prognosis associated with HPV–positive oropharyngeal cancer,^27^ not adjusting for HPV status might have biased our estimates for the association between poor oral hygiene habits and the survival of the oropharyngeal cancer patients towards the null.

This study has several strengths. First, this is the first study from Asia to examine the association between oral hygiene and the survival of HNC patients. Our results together with those of the two previous studies suggest that the association between poor oral hygiene and the worse survival of HNC patients may be universal across racial/ethnic groups, although more studies are needed to confirm this. Second, in addition to analyzing each oral hygiene habit separately, we used a composite score of oral hygiene, which consisted of tooth brushing, use of dental floss, and regular dental visits. Using a composite score of oral hygiene may better capture the oral hygiene practice of an individual. Finally, our study was the first to evaluate the gene–environment interaction on the relationship between oral hygiene and the survival of HNC patients. By examining the interaction between oral hygiene and the SNPs of *TLR2* and *TLR4*, our results suggested that poor oral hygiene may confer a poorer prognosis of HNC patients through the inflammatory pathways.

In conclusion, the current study found a worse OS of HNC patients associated with poor oral hygiene. This relationship was modified by the SNP, *TLR4 *rs11536889, suggesting the role of inflammatory pathways. Due to the limited number of studies, more investigations are needed to confirm these findings. Furthermore, more studies are warranted to determine the biological mechanisms explaining the influence of oral hygiene on the prognosis of HNC patients.

## CONFLICT OF INTEREST

None declared.
