# Supplementary figures and images for: Oral hygiene and the overall survival of head and neck cancer patients
Source: Cancer Med. 2019 Mar 13;8(4):1854–64. doi: 10.1002/cam4.2059 (PMC6488153; doi:10.1002/cam4.2059)

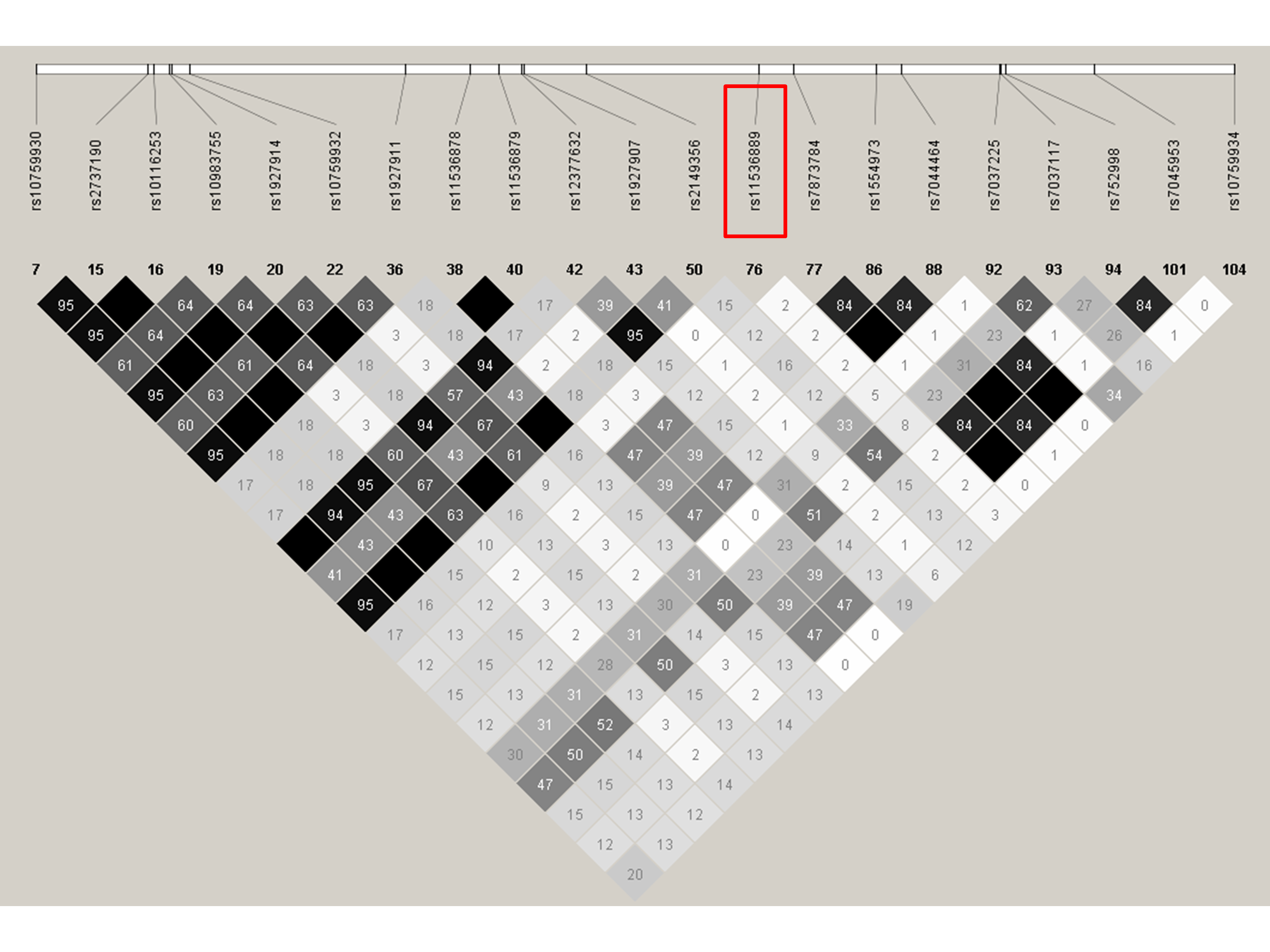

Supplement: Supplementary file 1 [file CAM4-8-1854-s001.tif]
