# Supplementary material for: Oral hygiene and the overall survival of head and neck cancer patients
Source: Cancer Med. 2019 Mar 13;8(4):1854–64. doi: 10.1002/cam4.2059 (PMC6488153; doi:10.1002/cam4.2059)
Supplement: Supplementary file 2 [file CAM4-8-1854-s002.doc]

Supplementary Table 1. The association between regular dental visits and the stage of head and neck cancer

|  | Regular dental visits | | |
| --- | --- | --- | --- |
|  | No  n (%) | Yes  n (%) | P^a^ |
| **Overall Stage** |  |  |  |
| 1+2 | 270 (42.0) | 30 (48.3) | 0.33 |
| 3+4 | 373 (58.0) | 32 (51.6) |  |
| **T stage** |  |  |  |
| 1+2 | 372 (58.4) | 52 (83.9) | <0.0001 |
| 3+4 | 265 (41.6) | 10 (16.1) |  |
| **N stage** |  |  |  |
| 0 | 366 (57.4) | 35 (56.5) | 0.89 |
| 1+2+3 | 272 (42.6) | 27 (43.5) |  |
